# Smartphone-based objective monitoring in bipolar disorder: status and considerations

**DOI:** 10.1186/s40345-017-0110-8

**Published:** 2018-01-23

**Authors:** Maria Faurholt-Jepsen, Michael Bauer, Lars Vedel Kessing

**Affiliations:** 1 Psychiatric Center Copenhagen, Rigshospitalet, Blegdamsvej 9, 2100 Copenhagen, Denmark; 2 Department of Psychiatry and Psychotherapy, Universitätsklinikum Carl Gustav Carus, Technische Universität Dresden, Dresden, Germany

## Abstract

In 2001, the WHO stated that: “The use of mobile and wireless technologies to support the achievement of health objectives (mHealth) has the potential to transform the face of health service delivery across the globe”. Within mental health, interventions and monitoring systems for depression, anxiety, substance abuse, eating disorder, schizophrenia and bipolar disorder have been developed and used. The present paper presents the status and findings from studies using automatically generated objective smartphone data in the monitoring of bipolar disorder, and addresses considerations on the current literature and methodological as well as clinical aspects to consider in the future studies.

## Introduction

Lifelogging, referring to the activity of producing a continual record of everyday life, has received great interest in the recent years. However, the idea of capturing detailed daily activities is not new. Almost 500 years ago, professor and physician Santorio Santorio (1561–1636) introduced a quantitative approach to medicine. He invented the thermometer and started to study the human metabolism in an objective way. For 30 years, he tracked and weighed all of his input (food and liquids) and output (urine and feces) on a daily basis, and to track his weight and metabolism in a detailed, objective and fine-grained manner, he invented a ‘weighing chair’. Looking at today’s quantification activities, many people use electronic devices for ‘lifelogging’/‘body-hacking’/‘self-quantification’/‘self-tracking’ with detailed and continuous tracking of physical and mental status, and is generally viewed as an empowering tool with “self-knowledge through self-tracking with technology”. Electronic self-tracking using computers began in the 1970s, and in 1994, Steve Mann, Professor, Ph.D. and known for his extensive work on computational photography, continuously transmitted his everyday life and activities 24 h 7 days a week using a chest-worn camera. Interest and opportunities for automatic and objective sensing of physical activity and dietary intake began to increase around 2002 (e.g., Actigraph, Polar, Fitbit), and in 2007, editors from the Wired Magazine established the Quantified Self communities—a worldwide collaboration between users and toolmakers who share an interest in self-knowledge and self-care through self-tracking. However, during recent years following the digital era, characterized by increased speed and breadth of knowledge turnover and a constant-on connectivity, considerations such as the balance between empowerment and surveillance, implications for autonomy, social and economic implications, cultural implications, stress-inducing consequences, personal privacy and ethical issues have been addressed and discussed (Selke 2016; Sharon 2017).

Within health care, e-health overall has been defined as health practice supported by any kind of electronic processes and communication and it dates back to 1999 (Oh et al. 2005; Yellowlees et al. 2011; Lal and Adair 2014). Mobile health (mHealth), on the other hand, has been defined as health services delivered specifically by mobile devices (e.g., mobile phones, PDAs, tablets and other wireless devices) (WHO 2011; Anthes 2016). In 2001, the WHO stated that: “The use of mobile and wireless technologies to support the achievement of health objectives (mHealth) has the potential to transform the face of health service delivery across the globe” (WHO 2011). Different mHealth interventions and monitoring systems have been developed and used within various medical conditions such as diabetes, asthma, cardiovascular disease, hypertension, chronic obstructive lung disease and headache (Hanlon et al. 2017). Within mental health, interventions and monitoring systems for depression, anxiety, substance abuse, eating disorder, schizophrenia and bipolar disorder have been developed and used (Riper et al. 2011; Beintner et al. 2012; Richards and Richardson 2012; Donker et al. 2013; Mayo-Wilson et al. 2013; Faurholt-Jepsen et al. 2015a; Berrouiguet et al. 2016; Berry et al. 2016; Depp et al. 2015; Hubley et al. 2016).

Based on the existing literature within the area, the aim of this paper is to discuss and emphasize several considerations and important aspects to consider in future studies within the area of using smartphones for passive and objective monitoring in bipolar disorder. Other systematic reviews on electronic subjective self-monitoring and internet-based treatment interventions within bipolar disorder have been published (Faurholt-Jepsen et al. 2016a; Dogan et al. 2017; Hidalgo-Mazzei et al. 2015b), thus the focus on the present article is objective monitoring using smartphones.

## Objective smartphone data and bipolar disorder

Bipolar disorder is a common and complex illness with an estimated prevalence of 1–2% and accounts as one of the most important causes of disability worldwide (Pini et al. 2005). In clinical practice, there are major challenges in diagnosing and treating bipolar disorder (Kupfer et al. 2015). Regarding clinical diagnosis, patients with bipolar disorder are often misdiagnosed and there is a delay in correct diagnosis from illness onset of several years (Kessing 2005). Clinical rating scales, such as the Hamilton Depression Rating scale (Hamilton 1967) and the Young Mania Rating scale (Abbs et al. 2012) are often used for the assessment of symptoms’ severity (state). Thus, the diagnostic process as well as the clinical assessment of severity of depressive and manic symptoms relies on subjective information and clinical evaluations raising issues including patient recall bias, decreased illness insight during affective episodes, and differences in clinical assessment experience. Therefore, objective methods for diagnosis and monitoring of illness activity are warranted.

There is a high rate of smartphone ownership worldwide, and it has been estimated that by the year of 2018, more than 2.5 billion people will own and use a smartphone (http://www.statista.com). Smartphones as a monitoring tool allow for ecological momentary assessments where real-time, time-stamped and fine-grained data are collected during naturalistic settings with a low level of intrusiveness (Shiffman et al. 2008; Wenze and Miller 2010; Aan het Rot et al. 2012; eMarketer 2016; Torous et al. 2017). Furthermore, using smartphones for monitoring allows for collection of automatically generated objective daily data (e.g., the number of text messages, the number of phone calls, GPS data, voice features) reflecting behavioral activities (passive objective monitoring), which may be related to psychopathology, that would not be easily accessible otherwise and by other electronic devices—e.g., data on phone usage, mobility, social activity and voice features. In this way, data on behavioral aspects can be collected during long-term using smartphones without the need for patients to interact with any self-monitoring app minimizing the level of obtrusiveness and the risk of fatigue.

Within mental health few studies using smartphones for collection of automatically generated objective data in patients with major depressive disorder (Robempath.pdf. 2017; Dang et al. 2016; Burns et al. 2011; Viewcontent.pdf. 2017) and schizophrenia (Ben-Zeev et al. 2017; Zhang et al. 2016) have been published. These studies mainly consist of feasibility studies or case reports, and the specificity of automatically generated objective smartphone data compared with healthy control individuals or the validity compared with clinically assessed symptoms using clinical rating scales has been sparingly investigated.

In bipolar disorder, changes in mood are accompanied by observable shifts in energy, activity, sleep and other behavioral aspects that may be quantified (Kupfer et al. 1974; Goodwin and Jamison 1996), and smartphones are able to automatically collect continuous data on some of these behavioral aspects. Within bipolar disorder research, more studies using smartphones for passive and unobtrusive monitoring of behavioral aspects as markers of trait and state have been published.

### Automatically generated objective smartphone data and clinically assessed symptoms

Several studies investigating the correlation between automatically generated objective smartphone data (e.g., the number of text messages, the number of phone calls, GPS data, voice features) and clinically assessed severity of depressive and manic symptoms assessed using the Hamilton Depression Rating Scale (Hamilton 1967) and the Young Mania Rating Scale (Young et al. 1978) and classification accuracy of automatically generated objective smartphone data and clinically assessed depressive and manic states have been published (Osmani 2015; Grünerbl et al. 2012, 2015; Beiwinkel et al. 2016; Karam et al. 2014a; Faurholt-Jepsen et al. 2014a, 2015b, 2016b, c; Gideon et al. 2016; Alvarez-Lozano et al. 2014; Vanello et al. 2012; Maxhuni et al. 2016; Guidi et al. 2015; Muaremi et al. 2014).

Studies employing overall regression models found that automatically generated smartphone data such as the number of text messages, the number and duration of phone calls, location/mobility data, voice features extracted during phone calls correlated with the level of depressive and manic symptoms assessed using the Hamilton Depression Rating Scale (Hamilton 1967) and the Young Mania Rating Scale (Young et al. 1978) were able to classify clinically assessed depressive and manic states (Beiwinkel et al. 2016; Faurholt-Jepsen et al. 2014a, 2015b, 2016b, c; Gideon et al. 2016; Karam et al. 2014b). Based on these studies, it seems that automatically generated objective smartphone data may represent an objective marker in bipolar disorder. Several limitations to the current literature and consideration regarding future studies are presented below.

Notably, some of the papers including automatically generated objective smartphone data and clinically assessed depressive and manic symptoms only presented case studies, employed individual patient analyses and did not present results from overall regression models (Osmani 2015; Grünerbl et al. 2012, 2015; Alvarez-Lozano et al. 2014; Maxhuni et al. 2016; Guidi et al. 2015; Muaremi et al. 2014).

### Automatically generated objective smartphone data and self-monitored symptoms

Within the area of bipolar disorder research, studies investigating the correlation between automatically generated objective smartphone data and smartphonebased self-monitored (by the patients) severity of depressive and manic symptoms have been published (Alvarez-Lozano et al. 2014; Abdullah et al. 2016; Palmius et al. 2016). The studies used various self-monitoring scales (e.g., VAS scales, standardized questionnaires, other scales), and thus it is difficult to compare findings from the studies. Overall, the studies found that several of the automatically generated objective smartphone data (e.g., location, app usage) correlated with the level of self-monitored depressive symptoms and manic symptoms as well as a self-assessed social rhythm score. One of the studies only employed individual patient analyses and did not present results from overall regression models (Alvarez-Lozano et al. 2014). Based on these few studies, it seems that it is not possible to conclude on the use of automatically generated objective smartphone data as a marker of smartphone-based patient-monitored illness activity in bipolar disorder. Several limitations to the current literature are presented below.

The validity of electronic self-monitored depressive and manic symptoms compared with clinically assessed depressive and manic symptoms has been addressed by the authors in a systematic review (Faurholt-Jepsen et al. 2016a), and is not the focus of the present paper.

## Considerations and future studies

### Considerations on the current literature

Given the current existing literature within the field of using smartphones for automatic and objective monitoring within bipolar disorder, several methodological as well as clinical considerations regarding the use of smartphones in future studies and implementation in clinical practice could be addressed.

Overall, results regarding the use of automatically generated objective smartphone data in bipolar disorder are based on individual studies with several methodological and clinical challenges and risk of bias at different levels.

Most of the published studies were pilot studies collecting data during quite short monitoring periods ranging between 4 weeks to 12 months and included rather small samples of patients with bipolar disorder ranging between 1 and 37 patients. Furthermore, the included patients presented with rather low levels of depressive and manic symptoms during the monitoring periods and thus, the correlation between automatically generated objective smartphone data and severe depressive and manic symptoms and episodes was not investigated. Along this line, the validity of automatically generated objective smartphone data compared with self-monitored depressive and manic symptoms may be affected by a decreased illness insight during affective episodes. Regarding the specificity of automatically generated objective smartphone data, only one study (Palmius et al. 2016) included a group of healthy control individuals, but did not investigate the diagnostic specificity of automatically generated smartphone data comparing the levels of automatically generated objective smartphone data between healthy control individuals and patients with bipolar disorder. The specificity compared with other mental disorders, healthy control individuals or healthy individuals at risk of bipolar disorder has not been investigated and is unknown.

Looking at the current literature, generally there was a lack of information provided on the selection process of the included and excluded patients, and thus an evaluation of selection bias was compromised. Furthermore, information on how many patients using iPhones (not allowing for collection of automatically generated objective smartphone data) that were asked to participate, but not included due to smartphone ownership was lacking in most studies. Patients owning and using iPhones may represent a group of patients presenting with a different course of illness than non-iPhone users.

The published studies used different self-monitoring scales, different clinical rating scales, different definitions of affective states, applied different duration criteria for affective states, and used different diagnostic systems (DSM-IV, ICD-10). Thus, this makes it difficult to compare results across studies.

Regarding detection bias, most of the studies did not state whether the clinicians collecting outcome data or patients conducting self-monitoring were blinded to the automatically generated objective smartphone data, and information on the clinicians’ rater experience was lacking.

Importantly, very few studies addressed possible confounding factors such as age and gender in the employed statistical analyses (Faurholt-Jepsen et al. 2014a, 2015b, 2016c), some studies presented only individual patient analyses (Osmani 2015; Grünerbl et al. 2012, 2015; Alvarez-Lozano et al. 2014; Maxhuni et al. 2016; Guidi et al. 2015; Muaremi et al. 2014), and few stated whether the statistical analyses were planned and specified in advance. Overall, most studies were at risk of bias at several levels.

### Future considerations

Overall, replication studies including larger sample sizes of patients monitoring symptoms and automatically generated objective smartphone data for prolonged periods of time are needed to validate previous study findings. Furthermore, designing observational studies using high methodological rigor both during the design phase, but also during the analysis phase are needed. In bipolar disorder research, future studies using automatically generated objective smartphone data, or combination of these, for illness monitoring may lead to detection of new trait and state markers (Torous et al. 2017; McIntyre et al. 2014). Future studies investigating the use of combined automatically generated smartphone data (and other biological and clinical measures) as a marker of risk, a marker of diagnosis, a marker of state, a marker of stage, a marker of treatment response and a marker of prognosis (McIntyre et al. 2014; Davis et al. 2015) would be groundbreaking. Smartphones are able to automatically collect ‘big data’, which are characterized by a large amount of complex data that are quickly generated and presents with great variety. This could provide opportunities for observation, exploration and hypothesis generation (Laney 2001; Glenn and Monteith 2014; Torous and Baker 2016; Monteith et al. 2015, 2016; Hidalgo-Mazzei et al. 2016a), but analyzing large amounts of data will require close collaboration between partners from diverse areas of expertise, such as researchers, clinicians, statisticians, and engineers. Furthermore, considering, planning and documenting the statistical analyses in advance, including predefined considerations on which potential confounding factors to include in the statistical analyses and how to account for multiple comparisons should be a priority. Also, to be able to evaluate the impact of individual confounding factors, the statistical analyses should be reported in both unadjusted and adjusted analyses.

Encouraging researcher to publish study protocols, regardless of study design, specifying all phases of the studies could assist in this process and allow future reviewers of a manuscript to be able to evaluate the presented findings more accurately.

The regulation of smartphone-based monitoring is opaque, and whether or not a smartphone-based monitoring system should be considered a medical device or not is unclear. However, the main concern to address continuously when using smartphones for monitoring and treatment in bipolar disorder should be the patients’ safety. Safety of data storage and encryption need careful considerations. Furthermore, ethical issues concerning providing and obtaining adequate informed content from participants, information quality in monitoring apps, personal privacy, and legal and cultural differences between nations are just some of the many important factors that needs to be addressed continuously when including patients in studies using smartphones for monitoring (Torous et al. 2017; Bauer et al. 2017).

Although the present paper concerns the use of smartphones for objective monitoring in bipolar disorder, it should be emphasized that currently there is a lack of randomized controlled trials investigating the possible positive, negative, and neutral effects as well as economical evaluations of smartphone-based treatment interventions (Faurholt-Jepsen et al. 2015a; Depp et al. 2015; Bilderbeck et al. 2016).

Finally, other studies have investigating the feasibility, usefulness and adherence of different smartphone-based monitoring systems (Bardram et al. 2012; Hidalgo-Mazzei et al. 2016b; Saunders et al. 2017), but have not investigated the validity, sensitivity and specificity of automatically generated objective smartphone data. Interestingly, protocols on studies including collection of automatically generated objective smartphone data and clinically rated symptoms have been published and are currently ongoing (Ritter et al. 2016; Hidalgo-Mazzei et al. 2015a; Faurholt-Jepsen et al. 2014b, 2017). Thus, future studies may hopefully be able to clarify some of the issues addressed in this paper.

There is a relevant diversity of smartphones on the market in which there are embedded diverse and dissimilar sensors and permissions to capture passive information (for instance, the iOS has more restrictions than the Android operating system). To overcome this issue, research groups around the world using smartphones for objective monitoring in bipolar disorder are trying to communicate with IT companies regarding getting access to uniform detailed automatically generated objective smartphone data in future studies. Hopefully, research groups will be able to collect the same passive information and amount of automatically generated smartphone data from all the operating systems. However, technical assistance to ensure uniform analyzable datasets of accurate information from which it would be possible to extract clinically meaningful conclusions will be necessary.

## Conclusion

Based on the current published literature on automatically generated smartphone data, it is still too early to evaluate which of the automatically generated objective smartphone data or combination of these that best correlate with levels of depressive or manic symptoms or classify affective states. Despite the seemly appeal and ease of use of automatically generated objective smartphone data in the monitoring of illness activity in bipolar disorder, further studies of high methodological rigor including larger samples of patients presenting with severe depressive and manic symptoms addressing confounding factors in the analyses are needed before being implemented as a standard monitoring tool.
